# Individual and combined associations of alanine aminotransferase and hemoglobin with metabolic syndrome in the elderly in Qingdao, China

**DOI:** 10.3389/fmed.2023.1152747

**Published:** 2023-08-09

**Authors:** Li Liu, Yuhan Shao, Enqiang Feng, Zhugang Shao, Dongming Xing

**Affiliations:** ^1^Cancer Institute, The Affiliated Hospital of Qingdao University, Qingdao University, Qingdao, Shandong, China; ^2^Qingdao Cancer Institute, Qingdao, Shandong, China; ^3^Qingdao Municipal Center for Disease Control and Prevention, Qingdao, Shandong, China; ^4^Qingdao Institute of Preventive Medicine, Qingdao, Shandong, China; ^5^Shandong Muhua Medical Technology Co., Ltd., Qingdao, Shandong, China; ^6^School of Life Sciences, Tsinghua University, Beijing, China

**Keywords:** alanine aminotransferase, hemoglobin, metabolic syndrome, elderly, combined association

## Abstract

**Background and aims:**

Combined associations of alanine aminotransferase (ALT) and hemoglobin (Hb) with metabolic syndrome (MetS) have not been assessed yet. The current study investigated the independent and combined relationships between ALT, Hb, and MetS in the elderly.

**Methods:**

The 37,966 elderly participants aged 65 years and older were recruited from community centers in Qingdao, China. The sampled elderly population visited the health centers once a year where they were offered a free health checkup. Based on a combination of ALT and Hb levels categorized by tertile, participants were grouped into nine groups (Group 1–9). Logistic regression models were used to analyze the individual and combined associations of ALT and Hb with MetS.

**Results:**

ALT and Hb were both independently related to MetS in both genders. With the elevation of ALT or Hb levels, risks for MetS and its components increased. Compared to the reference group (the 1st tertiles of both ALT and Hb levels), respective odds ratio of combined ALT and Hb for MetS in Group 2–9 ranged from 1.32–3.38 and 1.14–2.31 in men and women after adjusting for age, sex, education, married status, current smoking, current drinking, physical activity, and diet habit.

**Conclusion:**

ALT and Hb were both independently related to MetS and its components. Combined ALT and Hb levels could increase risks of MetS and its components than an elevation in ALT or Hb alone.

## Introduction

Metabolic syndrome (MetS) is a cluster of risk factors, including hypertension, hyperglycemia, dyslipidemia, and visceral obesity (1). MetS could result in cardiovascular disease, diabetes and high risk for all-cause mortality (2, 3). MetS has become a serious public health problem all over the world. It is estimated that one-quarter of the world population have MetS and its prevalence is expected to increase to 53% in 2035 (4, 5). According to the definition of the International Diabetes Federation, the reported prevalence of MetS in China was 25.59% in 2017 among individuals aged from 18 to 79 years (6). Furthermore, the prevalence of MetS by the National Cholesterol Education Program Adult Treatment Panel III definition among Chinese adults aged ≥60 years was 43.2% for men and 61.9% for women in 2017 (7). As a result, it is urgent to explore the etiology of MetS in the elderly for an early identification and intervention.

Most previous studies revealed that there was a positive association between Hb and MetS in both genders (8–10). The significant impact of Hb on MetS was observed only in men in a study conducted in Japan, while the Thailand study found the risk of MetS increased across successive quartiles of Hb only in women (11, 12). Besides, a case–control study suggested that Hb concentrations were not related to MetS (13). Thus, the conclusions about the association Hb and MetS between were in debate.

ALT is an indicator of non-alcoholic fatty liver disease (NAFLD), while NAFLD is regarded as the hepatic manifestation of MetS and has the same risk factors with MetS (14). Elevated ALT level, even within the normal range, was linked to prevalence of MetS (15). Other studies have also reported the significant impact of ALT level on MetS in different populations (15–17). However, several other studies showed that there was no relationship between ALT and MetS (18, 19). As a result, the association between ALT and MetS needs to be further clarified.

Because Hb and ALT both affect MetS through insulin resistance (20–22), combined relationships between Hb, ALT and MetS might exist. Up to now, no study has analyzed the combined linkage between Hb, ALT and MetS. Therefore, our study aimed to investigate the individual and combined associations of Hb and ALT with MetS based on data from the national basic public health service (BPHS) project for residents aged 65 and older in Qingdao, China.

## Methods

### Subjects and design

China has implemented the BPHS project since 2009, which aimed to address the major health issues of Chinese citizens. Relying on the national BPHS project, free health examinations for residents aged over 65 years old were carried out in 2021 in five community centers in Qingdao, China. Services of the elderly health examinations in 2021 in the current study included: 1. Questionnaire survey: Socio-demographic data, lifestyle habits such as smoking, alcohol consumption, physical activity, diet habit and family history of disease; 2. Anthropometric measurements: Height, weight, waist circumference (WC) and blood pressure. All participants wore light clothes and removed their shoes for height and weight assessment. Blood pressure was measured using a mercury sphygmomanometer and three consecutive blood pressure readings from the upper right arm of each participant were recorded at least 5 min apart. The mean of the three readings was recorded. WC was measured at the minimal abdominal girth between the costal margin and iliac crest with an inelastic cord in standing position; 3. Blood sample test: Hb levels were analyzed within 6 h using the hematology autoanalyzer DASIT SE 9000 (Sysmex Corporation, Kobe, Japan). The glucose oxidase method was used to measure fasting plasma glucose (FPG) (Olympus-AU, Olympus Co., Tokyo, Japan). Commercial enzymatic tests (Roche Diagnostics) were used for determining ALT, serum total cholesterol (TC), triglycerides (TG), and high-density lipoprotein cholesterol (HDL-C) concentrations. All blood samples were obtained from participants after 12-h fasting and after resting for at least 15 min.

Informed written consent was obtained from each participant. The study was conducted in accordance with the declaration of Helsinki and approved by the ethic committee of Qingdao Municipal Center for Disease Control and Prevention.

Among the individuals who participated in the health examination in 2021, those who had incomplete information on Hb, WC, FPG, TC, TG, HDL, LDL, systolic blood pressure (SBP), and diastolic blood pressure (DBP) were excluded (*n* = 3,501). Those with blood pressure, blood glucose or blood lipid altering medications, alcohol intake, as well as medications that can alter liver enzymes (*n* = 527) were also excluded. Ultimately, data from 37,966 individuals were included in the present study.

### Variable measurements

A balanced diet referred to intake of adequate types of food and the portion of vegetables to meat ratio was appropriate. Body mass index (BMI) was calculated as weight in kg divided by height in meter squared (kg/m^2^). MetS was defined according to the criterion of the National Cholesterol Education Program, Adult Treatment Panel III (NCEP-ATP III) (23). Individuals were identified as having MetS if they had at least three of the following risk factors: (1) WC ≥ 90 cm (men) or ≥ 80 cm (women); (2) TG ≥ 150 mg/dL (1.7 mmol/L); (3) HDL-C < 40 mg/dL (1.04 mmol/L) (men) or < 50 mg/dL (1.3 mmol/L) (women); (4) SBP/DBP ≥130/85 mmHg, or treated hypertension; (5) FPG ≥ 5.6 mmol/L, or who were prescribed antidiabetic agents.

### Statistical analysis

All statistical analyses were performed using SPSS 20.0 (IBM, Chicago, IL, USA). Continuous variables were presented as mean ± standard deviation (SD), and categorical variables were presented as frequencies and percentages. Group differences were tested using t test for continuous variables with normal distribution and the Mann–Whitney U test or Kruskal–Wallis test for continuous variables with a skewed distribution. The χ^2^ test was used to compare differences between groups for dichotomous variables. As shown in Table 1, subjects were grouped into Group 1–9 based on a combination of Hb and ALT levels in the tertile, and Group 1 was defined as the control group. Individual and combined associations of Hb and ALT with MetS were analyzed by logistic regression models in both genders. Area under receiver operating characteristic (AUROC) was investigate the associations of Hb, ALT and MetS. A two-tailed *p* < 0.05 was considered as statistically significant.

**Table 1:** Nine study groups according to the combination of ALT and Hb levels in the tertiles.

	ALT levels	Hb levels
Group 1	First tertile	First tertile
Group 2	First tertile	Second tertile
Group 3	First tertilef	Third tertile
Group 4	Second tertile	First tertile
Group 5	Second tertile	Second tertile
Group 6	Second tertile	Third tertile
Group 7	Third tertile	First tertile
Group 8	Third tertile	Second tertile
Group 9	Third tertile	Third tertile

## Results

### General characteristics of the study population by sex

Among the total 37,966 participants aged over 65 years old, the prevalence of MetS was 53.20%. As shown in Table 2, men were more like to be a smoker than women, while women reported higher percentage of regular physical activity and balanced diet habit than men. However, men and women did not differ in WC, SBP, DBP, TG, FPG, Hb, and ALT values (*P*>0.05 for all comparisons).

**Table 2:** General characteristics of the study population by sex.

	Total (n = 37,966)	Men (n = 13,670)	Women (n = 20,729)	*p* value
Age (years)	*74.0 ± 5.4*	*74.0 ± 5.3*	*74.1 ± 5.4*	0.629
Education				<0.001
Low	*9,088 (23.9)*	*3,868 (24.8)*	*5,220 (23.3)*	
Intermediate	*26,671 (70.2)*	*10,627 (68.2)*	*16,044 (71.6)*	
High	*2,207 (5.9)*	*1,078 (7.0)*	*1,129 (5.1)*	
Current smoking	*4,597 (12.1)*	*1,925 (12.4)*	*2,672 (11.9)*	0.208
Current drinking	*4,629 (12.2)*	*1,969 (12.6)*	*2,660 (11.9)*	0.025
Married	*32,268 (85.0)*	*13,223 (84.9)*	*19,045 (85.0)*	0.709
Physical activity				<0.001
Never	*26,265 (69.2)*	*10,590 (68.0)*	*15,675 (70.0)*	
1–7 days/week	*2,334 (6.1)*	*952 (6.1)*	*1,382 (6.2)*	
≥7 days/week	*9,367 (24.7)*	*4,031 (25.9)*	*5,336 (23.8)*	
Balanced diet habit	*3,127 (8.2)*	*1,236 (7.9)*	*1,891 (8.4)*	<0.001
WC (cm)	88 ± 8	88 ± 8	88 ± 8	0.177
SBP (mmHg)	150 ± 21	149 ± 21	150 ± 21	0.397
DBP (mmHg)	82 ± 11	82 ± 11	82 ± 11	0.830
Hb (g/L)	*136 ± 15*	*136 ± 15*	*136 ± 15*	0.618
TG (mmol/L)	1.56 ± 1.06	1.55 ± 1.00	1.57 ± 1.00	0.253
HDL (mmol/L)	1.49 ± 0.31	1.49 ± 0.31	1.50 ± 0.31	0.282
FPG (mmol/L)	6.52 ± 2.00	6.53 ± 2.00	6.52 ± 2.00	0.792
ALT (U/L)	23 ± 12	23 ± 12	23 ± 12	0.630

Data were presented as mean±SD for continuous variables, and *n* (%) for categorical variables. *p values in *t* tests for differences in means or Chi-square tests for differences in proportions.

### Independent association of ALT and Hb levels with MetS and its components

Table 3 showed the relationship between ALT and having MetS and other co-morbid conditions in men and women, respectively. In both genders, elderly participants with ALT levels in the second and third tertile were more likely to have central obesity, hyperglycemia, hypertension, hypertriglyceridemia and MetS (*p* < 0.05 for all comparisons) compared to those who were in first tertile after adjusting for age, sex, education, married status, current smoking, current drinking, physical activity, and diet habit, and similar associations were also found between Hb and MetS and its components. The independent relationships between Hb and MetS in different genders were shown in Table 4.

**Table 3:** Odds ratio (95% confidence interval) for individual association of ALT with MetS and its components in men and women.

	Men					Women				
	First tertile	Second tertile	*P* value	Third tertile	*P* value	First tertile	Second tertile	*P* value	Third tertile	*P* value
ALT (U/L)	<17		17–24		≥24		<17	17–24		≥24
Central obesity										
Unadjusted odds ratio	Ref.	1.28 (1.17–1.39)	<0.001	1.79 (1.65–1.93)	<0.001	Ref.	1.31 (1.19–1.45)	<0.001	1.81 (1.63–2.01)	<0.001
Adjusted odds ratio*	Ref.	1.25 (1.15–1.36)	<0.001	1.73 (1.59–1.87)	<0.001	Ref.	1.32 (1.19–1.46)	<0.001	1.80 (1.62–1.99)	<0.001
Hyperglycemia										
Unadjusted odds ratio	Ref.	1.35 (1.24–1.46)	<0.001	1.89 (1.74–2.05)	<0.001	Ref.	1.32 (1.23–1.41)	<0.001	1.77 (1.65–1.89)	<0.001
Adjusted odds ratio*	Ref.	1.33 (1.23–1.38)	<0.001	1.88 (1.79–2.19)	<0.001	Ref.	1.33 (1.24–1.38)	<0.001	1.78 (1.64–1.99)	<0.001
High blood pressure										
Unadjusted odds ratio	Ref.	1.07 (0.96–1.19)	0.201	1.30 (1.17–1.45)	<0.001	Ref.	1.16 (1.06–1.27)	0.001	1.25 (1.14–1.37)	<0.001
Adjusted odds ratio*	Ref.	1.16 (1.01–1.24)	0.045	1.39 (1.25–1.55)	<0.001	Ref.	1.15 (1.04–1.26)	0.001	1.26 (1.13–1.38)	<0.001
Hypertriglyceridemia										
Unadjusted odds ratio	Ref.	1.41 (1.29–1.55)	<0.001	2.17 (1.99–2.36)	<0.001	Ref.	1.28 (1.19–1.38)	<0.001	1.98 (1.84–2.13)	<0.001
Adjusted odds ratio*	Ref.	1.40 (1.29–1.54)	<0.001	2.15 (1.98–2.35)	<0.001	Ref.	1.27 (1.18–1.37)	<0.001	1.97 (1.83–2.12)	<0.001
Low HDL										
Unadjusted odds ratio	Ref.	1.07 (0.86–1.34)	0.525	1.14 (0.92–1.41)	0.222	Ref.	0.99 (0.81–1.21)	0.915	1.05 (0.86–1.28)	0.604
Adjusted odds ratio*	Ref.	1.12 (0.90–1.40)	0.300	1.21 (0.98–1.50)	0.083	Ref.	1.05 (0.86–1.29)	0.631	1.15 (0.94–1.41)	0.165
MetS										
Unadjusted odds ratio	Ref.	1.54 (1.42–1.67)	<0.001	2.37 (2.19–2.57)	<0.001	Ref.	1.31 (1.23–1.40)	<0.001	2.00 (1.87–2.14)	<0.001
Adjusted odds ratio*	Ref.	1.53 (1.41–1.66)	<0.001	2.36 (2.18–2.56)	<0.001	Ref.	1.32 (1.23–1.41)	<0.001	2.19 (1.85–2.12)	<0.001

*Adjusted for age, sex, education, married status, current smoking, current drinking, physical activity, and diet habit.

**Table 4:** Odds ratio (95% confidence interval) for individual association of Hb with MetS and its components in men and women.

	Men					Women				
	First tertile	Second tertile	*P* value	Third tertile	*P* value	First tertile	Second tertile	*P* value	Third tertile	*P* value
Hb (g/L)	<130		131–143		≥143		<130	130–143		≥143
Central obesity										
Unadjusted odds ratio	Ref.	1.33 (1.23–1.44)	<0.001	1.59 (1.46–1.72)	<0.001	Ref.	1.25 (1.13–1.38)	<0.001	1.56 (1.40–1.73)	<0.001
Adjusted odds ratio*	Ref.	1.31 (1.21–1.42)	<0.001	1.56 (1.44–1.70)	<0.001	Ref.	1.23 (1.11–1.35)	<0.001	1.55 (1.39–1.73)	<0.001
Hyperglycemia										
Unadjusted odds ratio	Ref.	1.33 (1.23–1.44)	<0.001	1.36 (1.25–1.48)	<0.001	Ref.	1.17 (1.09–1.25)	<0.001	1.23 (1.15–1.32)	<0.001
Adjusted odds ratio*	Ref.	1.34 (1.23–1.45)	<0.001	1.40 (1.29–1.52)	<0.001	Ref.	1.18 (1.10–1.26)	<0.001	1.26 (1.17–1.35)	<0.001
High blood pressure										
Unadjusted odds ratio	Ref.	1.14 (1.03–1.27)	0.013	1.27 (1.14–1.42)	<0.001	Ref.	1.14 (1.05–1.25)	0.003	1.32 (1.21–1.45)	<0.001
Adjusted odds ratio*	Ref.	1.19 (1.07–1.33)	0.001	1.36 (1.22–1.52)	<0.001	Ref.	1.19 (1.09–1.30)	<0.001	1.42 (1.29–1.56)	<0.001
Hypertriglyceridemia										
Unadjusted odds ratio	Ref.	1.10 (1.01–1.20)	0.025	1.04 (0.95–1.13)	0.419	Ref.	1.03 (0.96–1.10)	0.439	0.99 (0.92–1.06)	<0.001
Adjusted odds ratio*	Ref.	1.12 (1.03–1.33)	0.007	1.14 (1.05–1.25)	0.003	Ref.	1.05 (0.98–1.13)	0.133	1.10 (1.02–1.18)	0.011
Low HDL										
Unadjusted odds ratio	Ref.	0.97 (0.91–1.13)	0.256	1.01 (0.97–1.16)	0.149	Ref.	0.99 (0.81–1.21)	0.915	1.05 (0.86–1.28)	0.604
Adjusted odds ratio*	Ref.	0.94 (0.90–1.14)	0.246	0.87 (0.70–1.07)	0.194	Ref.	1.05 (0.86–1.29)	0.631	1.15 (0.94–1.41)	0.165
MetS										
Unadjusted odds ratio	Ref.	1.34 (1.23–1.45)	<0.001	1.47 (1.35–1.59)	<0.001	Ref.	1.14 (1.07–1.22)	<0.001	1.27 (1.19–1.36)	<0.001
Adjusted odds ratio*	Ref.	1.36 (1.25–1.47)	<0.001	1.56 (1.43–1.69)	<0.001	Ref.	1.16 (1.09–1.25)	<0.001	1.36 (1.27–1.46)	<0.001

*Adjusted for age, sex, education, married status, current smoking, current drinking, physical activity, and diet habit.

**Figure 1:**
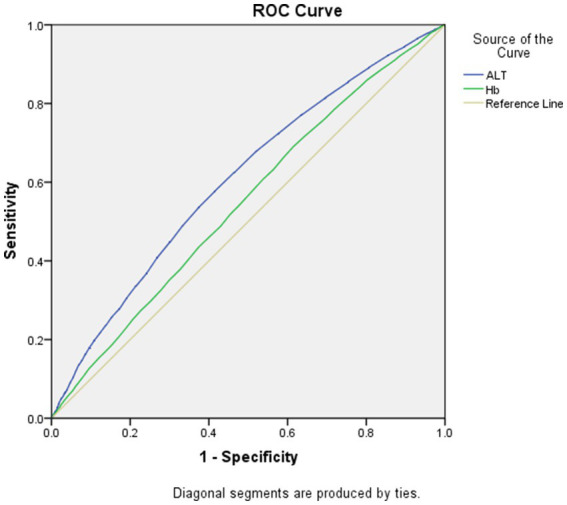
Area under the receiver operating characteristics curves of ALT and Hb for MetS in men.

**Figure 2:**
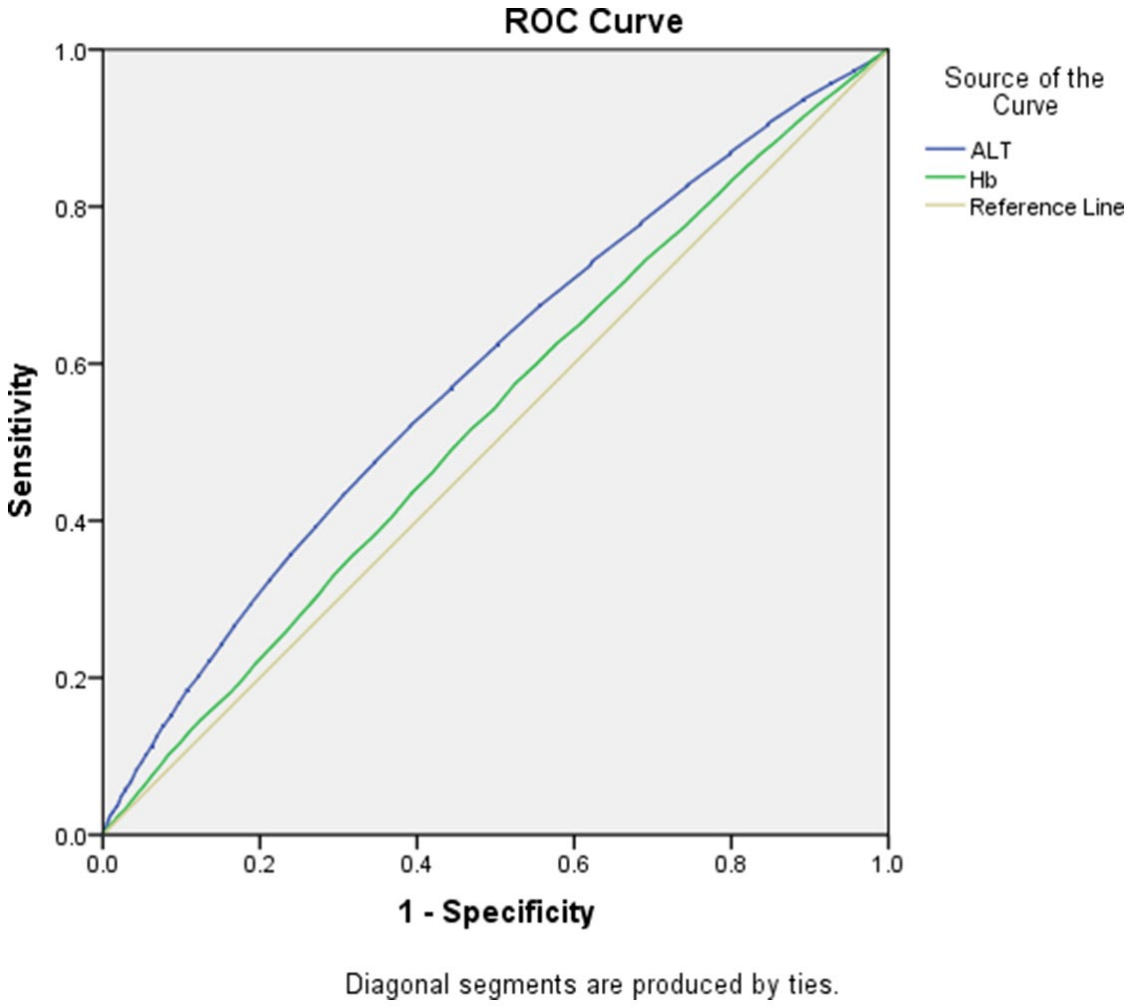
Area under the receiver operating characteristics curves of ALT and Hb for MetS in women.

As displayed in Figures 1, 2, the AUROCs of ALT and Hb for MetS were 0.61 (95%CI:0.60–0.62, *p* < 0.001), 0.55 (95%CI:0.54–0.56, *p* < 0.001) in men and 0.59 (95%CI:0.58–0.60, *p* = 0.004), 0.53 (95%CI: 0.52–0.54, *p* = 0.004) in women, respectively. The optimal cut-offs for ALT and Hb were 20.05 (sensitivity: 0. 58, specificity: 0.59), 131.50 (sensitivity: 0.69, specificity: 0.62) in men and 20.05 (sensitivity: 0.52, specificity: 0.61), 134.50 (sensitivity: 0.57, specificity: 0.53) in women, respectively.

### Combined associations of ALT and Hb with MetS and its components

As detailed in Tables 5, 6, compared with the first tertile of both ALT and Hb, the OR values of the groups increased with elevation of ALT and Hb levels in both genders. ALT combined with Hb was significantly correlated with central obesity, hyperglycemia, hypertension, hypertriglyceridemia and MetS in men and women. Men and women in Group 9 had 3.38-fold and 2.31-fold increased odds of having MetS than those in Group 1, respectively.

**Table 5:** Odds ratio (95% confidence interval) for combined ALT and Hb on MetS and its components in men.

	Group 1	Group 2	Group 3	Group 4	Group 5	Group 6	Group 7	Group 8	Group 9
Central obesity									
Unadjusted odds ratio	Ref.	1.42 (1.22–1.64)	1.37 (1.18–1.60)	1.28 (1.10–1.48)	1.60 (1.39–1.84)	1.88 (1.63–2.16)	1.71 (1.47–1.98)	2.09 (1.82–2.39)	2.72 (2.38–3.10)
Adjusted odds ratio*	Ref.	1.39 (1.20–1.61)	1.35 (1.16–1.57)	1.24 (1.07–1.44)	1.56 (1.35–1.79)	1.82 (1.57–2.09)	1.65 (1.42–1.91)	1.99 (1.74–2.29)	2.59 (2.26–2.96)
Hyperglycemia									
Unadjusted odds ratio	Ref.	1.22 (1.06–1.39)	1.30 (1.13–1.50)	1.36 (1.18–1.56)	1.74 (1.52–1.99)	1.57 (1.37–1.80)	1.73 (1.49–1.99)	2.36 (2.06–2.71)	2.37 (2.08–2.70)
Adjusted odds ratio*	Ref.	1.23 (1.08–1.41)	1.35 (1.17–1.56)	1.35 (1.18–1.56)	1.76 (1.53–2.01)	1.62 (1.41–1.86)	1.72 (1.49–1.99)	2.38 (2.07–2.73)	2.42 (2.12–2.77)
High blood pressure									
Unadjusted odds ratio	Ref.	1.27 (1.06–1.52)	1.23 (1.02–1.48)	1.19 (0.99–1.42)	1.13 (0.95–1.34)	1.39 (1.16–1.66)	1.26 (1.05–1.52)	1.51 (1.27–1.81)	1.64 (1.38–1.95)
Adjusted odds ratio*	Ref.	1.34 (1.12–1.60)	1.34 (1.11–1.62)	1.25 (1.04–1.50)	1.23 (1.04–1.46)	1.55 (1.29–1.86)	1.35 (1.12–1.63)	1.67 (1.40–2.00)	1.84 (1.54–2.20)
Hypertriglyceridemia									
Unadjusted odds ratio	Ref.	0.92 (0.79–1.08)	0.86 (0.73–1.01)	1.35 (1.16–1.58)	1.39 (1.19–1.60)	1.21 (1.04–1.41)	1.85 (1.59–2.15)	2.20 (1.91–2.53)	1.97 (1.72–2.26)
Adjusted odds ratio*	Ref.	0.95 (0.81–1.13)	0.95 (0.81–1.13)	1.33 (1.14–1.55)	1.41 (1.21–1.63)	1.33 (1.14–1.55)	1.80 (1.55–2.10)	2.21 (1.91–2.54)	2.12 (1.84–2.44)
Low HDL									
Unadjusted odds ratio	Ref.	0.63 (0.43–0.94)	0.84 (0.58–1.23)	1.19 (0.85–1.68)	0.66 (0.45–0.96)	0.86 (0.60–1.23)	1.08 (0.76–1.55)	0.96 (0.69–1.35)	0.85 (0.61–1.18)
Adjusted odds ratio*	Ref.	0.67 (0.45–1.19)	0.95 (0.65–1.39)	1.26 (0.89–1.77)	0.71 (0.49–1.04)	1.00 (0.69–1.44)	1.15 (0.80–1.65)	1.05 (0.75–1.48)	0.98 (0.70–1.38)
MetS									
Unadjusted odds ratio	Ref.	1.29 (1.11–1.50)	1.31 (1.12–1.52)	1.63 (1.41–1.89)	1.89 (1.64–2.18)	1.93 (1.67–2.22)	2.11 (1.82–2.45)	2.92 (2.55–3.36)	3.21 (2.81–3.67)
Adjusted odds ratio*	Ref.	1.32 (1.14–1.53)	1.40 (1.19–1.64)	1.62 (1.40–1.88)	1.94 (1.68–2.23)	2.06 (1.78–2.38)	2.09 (1.80–2.43)	2.96 (2.57–3.40)	3.38 (2.95–3.88)

*Adjusted for age, sex, education, married status, current smoking, current drinking, physical activity, and diet habit.

**Table 6:** Odds ratio (95% confidence interval) for combined ALT and Hb on MetS and its components in women.

	Group 1	Group 2	Group 3	Group 4	Group 5	Group 6	Group 7	Group 8	Group 9
Central obesity									
Unadjusted odds ratio	Ref.	1.19 (1.02–1.39)	1.48 (1.25–1.76)	1.30 (1.11–1.52)	1.59 (1.36–1.87)	1.80 (1.52–2.13)	1.71 (1.43–2.05)	2.05 (1.73–2.42)	2.61 (2.20–3.10)
Adjusted odds ratio*	Ref.	1.16 (0.99–1.36)	1.48 (1.24–1.76)	1.23 (1.05–1.44)	1.50 (1.28–1.77)	1.74 (1.47–2.06)	1.59 (1.33–1.91)	1.89 (1.59–2.24)	2.45 (2.05–2.92)
Hyperglycemia									
Unadjusted odds ratio	Ref.	0.99 (0.89–1.12)	1.09 (0.97–1.23)	1.21 (1.08–1.36)	1.45 (1.29–1.62)	1.40 (1.25–1.57)	1.55 (1.37–1.75)	1.91 (1.70–2.14)	1.93 (1.72–2.15)
Adjusted odds ratio*	Ref.	1.01 (0.90–1.13)	1.12 (0.99–1.26)	1.21 (1.08–1.36)	1.46 (1.30–1.63)	1.43 (1.27–1.60)	1.54 (1.36–1.74)	1.91 (1.71–2.15)	1.95 (1.75–2.19)
High blood pressure									
Unadjusted odds ratio	Ref.	1.03 (0.89–1.20)	1.23 (1.05–1.45)	1.09 (0.94–1.26)	1.28 (1.10–1.48)	1.39 (1.19–1.61)	1.10 (0.94–1.29)	1.34 (1.15–1.55)	1.54 (1.33–1.78)
Adjusted odds ratio*	Ref.	1.09 (0.94–1.26)	1.35 (1.15–1.58)	1.14 (0.98–1.32)	1.37 (1.18–1.59)	1.53 (1.32–1.79)	1.16 (0.99–1.36)	1.44 (1.24–1.67)	1.72 (1.48–1.99)
Hypertriglyceridemia									
Unadjusted odds ratio	Ref.	0.87 (0.77–0.99)	0.87 (0.76–0.99)	1.23 (1.09–1.39)	1.23 (1.09–1.39)	1.08 (0.96–1.22)	1.81 (1.60–2.05)	1.91 (1.70–2.14)	1.75 (1.56–1.96)
Adjusted odds ratio*	Ref.	0.91 (0.80–1.04)	0.99 (0.86–1.13)	1.22 (1.08–1.38)	1.27 (1.12–1.43)	1.20 (1.06–1.35)	1.80 (1.59–2.05)	1.94 (1.72–2.18)	1.94 (1.73–2.18)
Low HDL									
Unadjusted odds ratio	Ref.	0.68 (0.48–0.96)	0.71 (0.49–1.01)	1.03 (0.76–1.41)	0.77 (0.56–1.07)	0.61 (0.43–1.03)	1.04 (0.75–1.44)	0.83 (0.61–1.14)	0.75 (0.55–1.02)
Adjusted odds ratio*	Ref.	0.73 (0.51–1.03)	0.79 (0.55–1.15)	1.12 (0.82–1.53)	0.86 (0.62–1.19)	0.70 (0.49–1.05)	1.15 (0.83–1.60)	0.95 (0.69–1.30)	0.90 (0.65–1.24)
MetS									
Unadjusted odds ratio	Ref.	1.15 (1.04–1.32)	1.16 (1.03–1.30)	1.19 (1.07–1.34)	1.42 (1.28–1.59)	1.42 (1.27–1.59)	1.80 (1.59–2.03)	2.10 (1.88–2.35)	2.17 (1.95–2.42)
Adjusted odds ratio*	Ref.	1.14 (1.03–1.31)	1.25 (1.11–1.41)	1.20 (1.07–1.34)	1.46 (1.30–1.63)	1.52 (1.36–1.70)	1.79 (1.58–2.03)	2.13 (1.90–2.39)	2.31 (2.07–2.59)

*Adjusted for age, sex, education, married status, current smoking, current drinking, physical activity, and diet habit.

## Discussion

The present study that included 37,966 community individuals aged 65 years and older in Qingdao, China suggested that ALT and Hb were independently correlated with MetS and its components in both genders. Combined elevation of ALT and Hb levels could increase the risk of having MetS and its components than elevation of only one index. To the best of our knowledge, this is the first study to report the combined associations of ALT and Hb with MetS and its components.

Many studies have reported the association between ALT and MetS, but the conclusions were not consistent. Individuals with MetS had a significantly higher prevalence of unexplained elevations in ALT levels than in those without MetS in the US National Health and Nutrition Examination Survey (NHANES) (24). Participants with abnormal ALT levels had higher risk of having MetS when compared to those with normal ALT levels (OR = 2.02, *p* = 0.0046) (25). Besides, when normal ALT levels were divided into quartiles, the second, third and fourth quartile groups all had higher risks of having MetS (15). The normal upper limit of ALT has been set around 40 U/L since serum ALT was used as a surrogate marker for hepatitis (26, 27). In the current study, ALT was positively associated with MetS but it could not be an authentic predictor of MetS in both men and women. ALT levels in the second tertile group were in normal range, but participants in this group had higher risks for MetS and its components compared to the first tertile of ALT group. In line with previous studies, our study showed that even in the normal range, high ALT was still a risk factor of MetS and its components. Insulin resistance (IR) could explain the relationship between ALT and MetS. ALT was a surrogate index for NAFLD and its level could increase extensively in NAFLD. Individuals with NAFLD had IR in liver, adipose tissue and skeletal muscle (28). IR could result in the development of steatosis and fibrosis by enhancing fatty acid β-oxidation and oxidative stress (29), and then led to disturbances in lipid metabolism (30, 31). In addition, an inflammatory effect in the liver might be another factor resulting in MetS, which impaired insulin signaling and resulted in failure to inhibit glucose production (32, 33).

A cross-sectional study with 1,339 patients in Thailand showed that Hb concentration was significantly associated with MetS components in women but not in men (12). Interestingly, another study found the highest and third Hb concentration quartiles were associated with MetS in men but not in women when compared to the lowest Hb concentration quartile (11). Additionally, Hb was also reported to have no relation to MetS (13). The inconsistency of the association between Hb and MetS as reported in the literature may be due to differences in study design, ethnicity and grouping categorization. In our study Hb was associated with MetS both in men and women, but Hb predicting MetS was low accuracy. In fact, an Ethiopia study displayed that men in the third quartile of Hb concentrations had 2-fold increased odds for MetS compared with the lowest reference quartile, while women in the fourth Hb quartile had 2.37-fold increased odds of having MetS compared with the reference group (34). Other studies also described that high Hb level could increase risk of having MetS in both genders (8–10). However, the mechanism that high Hb level could lead to MetS remains unclear. Hematocrit caused by high Hb, as the foremost determinant of whole-blood viscosity, might lead to slow blood flow. Slow blood flow influenced the delivery of insulin, glucose, and oxygen to the tissue, which could result in IR (20). Moreover, accumulation of hematological components due to slow blood flow was reported to be a catalyst of MetS and its components (35, 36).

Up to now, no study has reported the combined associations of ALT and Hb with MetS. Our study showed that increased combined ALT and Hb was more significantly associated with MetS and its components than an increase in ALT or Hb alone in both genders. After adjusting for confounding factors, Men and women in Group 9 had 3.38-fold and 2.31-fold increased odds of having MetS than those in Group 1, respectively. Therefore, combined ALT and Hb might be considered as routine indicators to assess MetS in clinical practice.

There are some limitations to this study. First, the sample was large enough but not obtained from a random sample. Secondly, the cross- sectional study design could not demonstrate causal relation, therefore, studies are needed to verify the results of our study in the future. Thirdly, although several confounding factors were controlled in the current study, there might be residual confounding factors that were ignored in the analysis. Finally, ALT and Hb were only measured once, and high intra-individual variations might affect the results.

## Conclusion

ALT and Hb concentrations were independently associated with MetS and combined elevation of ALT and Hb levels could increase risks of MetS and its components than an elevation in ALT or Hb alone. Thus, it can be helpful in clinical settings to identify patients at risk of both high ALT and Hb levels, which is closely related to MetS.

## Data availability statement

The raw data supporting the conclusions of this article will be made available by the authors, without undue reservation.

## Ethics statement

The studies involving human participants were reviewed and approved by the ethic committee of Qingdao Municipal Center for Disease Control and Prevention. The patients/participants provided their written informed consent to participate in this study.

## Author contributions

LL, YS, ZS, and EF contributed to the manuscript composition, quality assessment, and records review. LL, YS, and DX designed the manuscript and analyzed the data. LL and DX were responsible for the integrity of this work and contributed to final study selection and manuscript review. DX coordinated the data acquisition and standardization. All authors reviewed and approved the final manuscript.

## Funding

This work was supported by grants from Qingdao Outstanding Health Professional Development Fund.

## Conflict of interest

ZS was employed by Shandong Muhua Medical Technology Co., Ltd.

The remaining authors declare that the research was conducted in the absence of any commercial or financial relationships that could be construed as a potential conflict of interest.

## Publisher’s note

All claims expressed in this article are solely those of the authors and do not necessarily represent those of their affiliated organizations, or those of the publisher, the editors and the reviewers. Any product that may be evaluated in this article, or claim that may be made by its manufacturer, is not guaranteed or endorsed by the publisher.
